# Coping Strategies as a Mental Health Protection Factor of Spanish Nurses during COVID-19

**DOI:** 10.3390/ijerph182312748

**Published:** 2021-12-03

**Authors:** María del Mar Molero-Jurado, María del Carmen Pérez-Fuentes, José Jesús Gázquez-Linares, Azucena Santillán García

**Affiliations:** 1 Department of Psychology, Faculty of Psychology, University of Almería, 04120 Almería, Spain; mmj130@ual.es (M.d.M.M.-J.); jlinares@ual.es (J.J.G.-L.); 2 Department of Psychology, Universidad Politécnica y Artística del Paraguay, Asunción 1628, Paraguay; 3 Department of Psychology, Universidad Autónoma de Chile, Providencia 7500000, Chile; 4 Hospital Universitario de Burgos, 09006 Burgos, Spain; ebevidencia@gmail.com

**Keywords:** perceived threat, coping strategies, COVID-19, nursing

## Abstract

Background: Due to the healthcare crisis caused by COVID-19, nurses have been exposed to stressful, uncertain situations. In such situations, emotional coping strategies are especially important due to their repercussion on health. The purpose of this study is analyze the relationships between nurses’ coping strategies and health, with attention to factors related to perceived threat and/or someone close to them is COVID-19 positive. Methods: This descriptive cross-sectional study was done with a sample of 351 nurses in Spain. In addition to the questionnaire on perception of threat from COVID-19, the Cognitive Emotion Regulation Questionnaire and the General Health Questionnaire, an ad hoc question asked them whether someone close to them was COVID-19 positive. Results: Perceived threat and use of negative coping strategies were related, and these strategies were related to a greater presence of somatic symptoms, anxiety, social dysfunction, and depression. Conclusions: Given the work and personal influence of coping on nurses, interventions must be designed to promote adaptive strategies.

## 1. Introduction

The healthcare crisis caused by the SARS-CoV-2 pandemic has placed healthcare services around the world at risk [1]. This has been a stress test for healthcare professionals, institutions, and governments, as well as for the general population [2]. Nurses have been subjected to unheard of situations in which they have been faced with a drastic increase in severe patients affected by COVID-19. Add to this the uncertainty of treating a microorganism that little is known about and insufficient individual protection equipment [3]. In Spain, this has caused a very high number of healthcare professionals to be tested COVID-19 positive [4].

In such a complex panorama, nurses have been obligated to face stressful situations maintained over months, and this could affect their mental health, requiring specific intervention [5,6,7,8,9,10]. In addition, stress and anxiety are related [11], so interventions for reducing them would have a positive effect on the mental health of persons affected, especially those professionals most involved [12].

Perceived threat to health is based on susceptibility and vulnerability perceived [13] and on states of prolonged hypervigilance, feelings of danger, and high sensitivity to appearance or recurrence of illness [14]. Perception of threat from COVID-19 is related to sadness–depression, anxiety, and hostility and anger, with a circular relationship in which perceived threat influences presence of negative mood state, which in turn promotes a feeling of threat [15,16]. Therefore, the level of perceived threat from COVID-19 could be related to the use of different cognitive emotion regulation strategies. It is also known that those affected by COVID-19 could feel anxiety and distress from the disease symptoms themselves, as well as fear of consequences, and those in quarantine may also be bored, lonely, and angry [17].

There is a generalized perception of threat from COVID-19, where risk factors—and therefore, more vulnerability to its effects—include being a woman, having children still minors in one’s care, and a lower education [18]. The fact that unknown problems are still emerging related to COVID-19 and its cause has produced uncertainty, and psychologically uncertain situations are usually accompanied by anxiety due to agitation from anticipation of threat and stress. Therefore, it must be managed to lower psychological distress and ensure adjustment [11,19,20]. Therefore, we may think that there are different profiles according to different characteristics within the COVID-19 context such as level of perceived threat and whether or not someone close is COVID-19 positive.

In addition, negative coping strategies, such as self-blame, rumination, catastrophism, and blaming others are closely related to alterations in health [16,21]. Meanwhile positive coping strategies are not [16] or are even related negatively to the presence of symptoms such as psychological distress, anxiety, stress, or sleeping problems [22,23]. In this sense, and according to the Cognitive Appraisal Theory by Lazarus and Folkman [24], stress is a two-way process. It involves the production of environmental stress factors and the response of the person subjected to those factors. In this theory, coping is defined as the efforts a person makes to anticipate, challenge, or change conditions to alter a situation that is appraised as stressful. Thus, coping strategies are cognitive, behavioral efforts that a person must make to be able to manage external (environmental, stressors) or internal (emotional state) demands that exceed the person’s resources [24,25]. In a stressful situation, people develop different ways of coping, which are related to personal factors, situational demands, and available resources, with the goal of reestablishing balance in the organism faced with reactions triggered by the stress factor [26,27]. It is worth mentioning that the type of coping strategy used in a concrete situation varies according to personality or experience, as well as the characteristics of the situation [28,29,30,31]. Hospital nurses tend to develop problem-focusing strategies, which protect them from stress [28]. Other authors have shown that the acquisition of skills is among the main adaptive coping strategies in the nursing work environment: training in relaxation for adequate use of emotions, biofeedback, meditation, physical training, or modifying cognitive processes [32,33].

Carver, Scheier, and Weintraub [34] suggested that it is unimportant whether a strategy is active or passive as long as it is useful and beneficial in successfully resolving a stressful situation. Thus, in stressful situations in clinical nursing practice which can and should be resolved immediately, such as solving an acute problem in the ICU (Intensive Care Unit), problem-focused or active coping strategies should be used. While in those situations where resolution is more uncertain that do not depend so much on nursing intervention, strategies focusing on emotion, or passive, would be considered adaptive. In this regard, the best coping strategies in critical settings (ICU) are associated with greater satisfaction and less secondary stress syndrome, while avoidance strategies predict secondary traumatic syndrome [35].

Furthermore, when the coping strategies used are inadequate, the individual shows physiological and behavioral alterations. This can lead to deterioration of the person’s health, which is manifested as cardiovascular alterations [36] or worsening of pre-existing pathologies, such as diabetes [37] as well as sleeping problems [17]. There are even signs of the pernicious effect of stress in some biological parameters of health professionals [38]. Nurses who have worked in COVID-19 units show higher levels of stress, exhaustion, and depressive mood as well as lower job satisfaction than their colleagues in the usual hospital units due especially to job tension and uncertainty about the future [39,40,41]. In addition, perceived threat can itself cause a diversity of psychological maladjustments [16]. Therefore, due to the various complicated situations nurses are faced in the context of COVID-19, the impact on their quality of life and health can be strong, and it is important to determine the best strategies for coping with stress in this discipline to avoid negative consequences. On this basis, we proposed to analyze the relationships between the use of coping strategies and health by nurses in the context of COVID-19 with attention to related factors such as perceived threat and/or whether or not someone close to them is COVID-19 positive.

## 2. Materials and Methods

### 2.1. Participants

A total of 505 nurses originally accepted to participate in the survey. After review, 154 cases had to be discarded, specifically because 148 questionnaires were incomplete and six cases were found to be answered at random by control questions inserted in the questionnaire. Thus, the study sample was made up of 351 nurses (Spain), aged 22 to 64, and a mean age of 40.91 (*SD* = 10.98). Of the total sample, 86% (*n* = 302) were women. They were also asked whether anyone close to them was COVID-19 positive, to which 62.7% (*n* = 220) of the participants answered affirmatively.

### 2.2. Instruments

Items on sex and age were included to collect sample descriptive data. They were also asked if anyone close to them was COVID-19 positive. The questionnaire on perception of threat from COVID-19 [42] was administered as a measure of perceived threat and worry about the disease. This instrument consists of five items rated on a 10-point Likert-type scale, from which a single dimension is found, where higher scores indicate greater perceived threat from COVID-19.

Cognitive Emotion Regulation Questionnaire (CERQ) [43]. In this study, the Spanish version of the questionnaire (CERQ-S) [44] was administered. It evaluates different strategies for coping with adverse situations in 36 items with five answer choices (from 1 “almost never” to 5 “almost always”). The questionnaire subscales and their reliability coefficients in this study were self-blame (ω = 0.68), acceptance (ω = 0.63), rumination (ω = 0.78), positive refocusing (ω = 0.87), planning (ω = 0.81), positive reappraisal (ω = 0.81), putting into perspective (ω = 0.64), catastrophizing (ω = 0.62), and other-blame (ω = 0.89).

General Health Questionnaire (GHQ-28) [45]. The Spanish version of the questionnaire [46] was applied. It evaluates four dimensions of mental health: somatic symptoms, anxiety and insomnia, social dysfunction, and depression. The GHQ-28 is composed of 28 items, with four answer choices. The Likert-type scale correction method was used, attributing a value of 0 to 3 to each answer choice. The reliability of the instrument is ω = 0.93 for the total scale, and for each of the subscales, the reliability values are as follows: somatic symptoms (ω = 0.86), anxiety and insomnia (ω = 0.90), social dysfunction (ω = 0.81) and depression (ω = 0.91).

### 2.3. Procedure

The study was descriptive and cross-sectional with snowball sampling. A CAWI (Computer-Aided Web Interviewing) survey was used for data collection. It was publicized on social networks and by instant messaging on 1–12 May 2020, that is, during the seventh and eighth weeks of confinement of the Spanish population. The participants filled out the tests individually in an estimated mean time of 10–15 min. Inclusion criteria were as follows: (1) nurses actively employed at the time of the survey and during the six previous months, and (2) nurses in care services who had direct contact with patients and their families. Participation was voluntary, and before beginning to answer the questionnaire, participants gave their informed consent by marking a box which then allowed them access to the questionnaire. To detect random answers, control questions were inserted in the questionnaire. This study was approved by the University of Almeria Bioethics Committee (Ref. UALBIO2020/032).

### 2.4. Data Analysis

First, correlation analyses were performed and descriptive statistics were calculated. For comparison of means, Student’s *t*-test was applied, with Cohen’s *d* [47] for estimating the effect size. Then, a two-stage cluster analysis was done to classify cases by the variables: perceived threat (dichotomized as low/high, taking the sample mean as the cutoff point and applying visual clustering) and somebody close who was COVID-19 positive (yes/no). For cluster comparison to detect if there were any significant differences with regard to the use of coping strategies and the health subscales, a multivariate analysis (MANOVA) was performed with IBM SPSS Statistics for Windows v. 24.0 (Armonk, NY: IBM Corp.) [48]. In addition, to examine the reliability of the instruments used for data collection, McDonald’s Omega was calculated, following the recommendations of Ventura-León and Caycho [49].

## 3. Results

### 3.1. Descriptive Analysis of Sociodemographic Variables and Use of Coping Strategies

When the associations between the various coping strategies and participant age were examined, negative correlations were observed for the acceptance (r = −0.11, *p* < 0.05), rumination (r = −0.16, *p* < 0.01), refocus on planning (r = −0.10, *p* < 0.05), positive reappraisal (r = −0.11, *p* < 0.05), and putting into perspective (r = −0.13, *p* < 0.05) strategies.

Table 1 presents the results of the comparison of means for coping strategies by gender. In general, there were no statistically significant differences between men and women. There was only a tendential difference in the positive reappraisal strategy, where women had a higher mean score.

### 3.2. Use of Coping Strategies by Nurses in the Context of COVID-19

Table 2 shows how perceived threat from COVID-19 correlated positively to self-blame, rumination, catastrophizing, and other-blame strategies and negatively with positive reappraisal.

In addition, depending on whether or not someone close to them was COVID-19 positive, significant differences were found in the use of rumination (t_(349)_ = 2.09, *p* < 0.05, d = 0.23) as a coping strategy, where those who said someone close was positive (M = 12.62, SD = 3.38) had a higher score than those who did not (M = 11.84, SD = 3.46).

### 3.3. Profiles by Someone Close COVID-19 Positive and Perceived Threat

**Figure 1:**
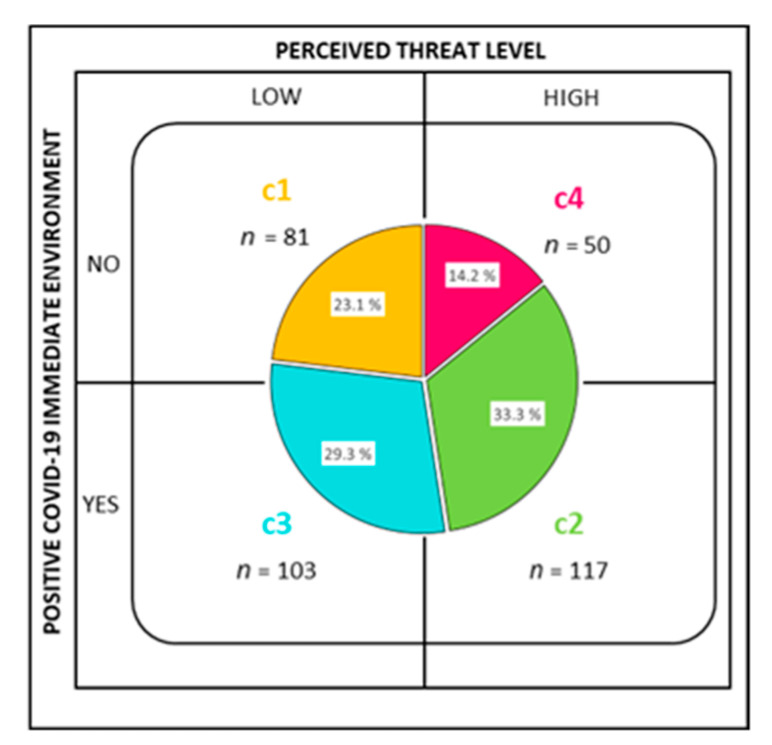
Profiles by someone close COVID-19 positive and perceived threat.

Figure 1 shows the results of the cluster analysis, where four groups were formed and classified by whether the level of perceived threat from COVID-19 was high or low (above or below the sample mean, respectively) and whether someone close was COVID-19 positive or not.

The cases in Cluster 1 (C1) have no COVID-19 positive cases close to them and perceive a low level of threat. Cluster 2 (C2) groups were those who said they did have positive cases close to them and perceived a high level of threat, unlike those in Cluster 3 (C3), who in spite of having positive cases near them perceived a low level of threat from COVID-19. Finally, Cluster 4 (C4) contained those with a high level of perceived threat from COVID-19, and nobody close to them was COVID-19 positive.

### 3.4. Coping Strategies and Health: Correlations and Cluster Comparison

As shown in Table 3, some of the coping strategies related positively to the presence of health problems. More specifically, catastrophism was associated positively with all of the GHQ-28 subscales. In other cases, such as rumination, self-blame, and blaming others, the most frequent association was with somatic symptoms, anxiety, insomnia, and also depression.

Concerning coping strategies with negative correlations, we found that putting into perspective was negatively associated with the presence of anxiety/insomnia, social dysfunction, and depression. Acceptance was negatively associated with social dysfunction, while refocus on planning was related to depression. Positive refocusing and positive reappraisal were associated negatively with all the dimensions of the GHQ-28.

The results of the multivariate analysis testing for between-group cluster differences in coping strategies and health are shown below.

The coping strategies in the groups were revealed to be significantly different (Wilk’s Λ = 0.803, F_(3, 347)_ = 2.86, *p* < 0.001, ηp^2^ = 0.07). The univariate analyses showed significant between-group differences in particular for rumination (F_(3, 347)_ = 5.16, *p* < 0.01, ηp^2^ = 0.04), catastrophizing (F_(3, 347)_ = 17.80, *p* < 0.001, ηp^2^ = 0.13), and other-blame (F_(3, 347)_ = 6.46, *p* < 0.001, ηp^2^ = 0.05). Rumination was higher in C2 than the rest of the groups, while catastrophizing was higher in C4, followed by C2, and it differed significantly from the rest, as observed in Table 4, where the descriptive statistics are shown for each cluster.

The multivariate analysis showed significant differences in the health subscales (Wilk’s Λ = 0.759, F_(3, 347)_ = 8.35, *p* < 0.001, ηp^2^ = 0.08). Furthermore, the univariate analyses showed significant differences between the groups for all the GHQ-28 subscales: somatic symptoms (F_(3, 347)_ = 25.55, *p* < 0.001, ηp^2^ = 0.18), anxiety/insomnia (F_(3, 347)_ = 26.63, *p* < 0.001, ηp^2^ = 0.18), social dysfunction (F_(3, 347)_ = 8.22, *p* < 0.001, ηp^2^ = 0.06), and depression (F_(3, 347)_ = 6.93, *p* < 0.001, ηp^2^ = 0.05). The professionals in C2 and C4 scored higher in somatic symptoms, anxiety/insomnia, social dysfunction, and depression than C1 and C3.

## 4. Discussion

After analyzing the relationships between nurses’ coping strategies and health based on related factors, threat perceived from COVID-19 showed significant associations with the use of different cognitive emotion regulation coping strategies. Previous studies have shown that negative coping strategies, such as self-blame, rumination, catastrophizing, and other-blame lead to health problems in nurses [16]. In our study, catastrophizing was shown to be the strategy most closely related to all the health subscales, while self-blame, rumination and other-blame showed a positive correlation with somatic symptoms, anxiety/insomnia, and depression. Rumination was found to be more related to someone close being COVID-19 positive, and considering that Thanoi and Klainin-Yobas [50] suggested that this was one of the determining factors in the mental health of nursing students, it would be of interest to assess this strategy in undergraduates to intervene appropriately and prevent secondary effects of rumination in registered nurses. In addition, “positive” coping strategies, such as acceptance, positive refocusing, planning, positive reappraisal, and putting into perspective were health protection factors [16,22].

COVID-19 has had a strong impact on the mental health of nurses, and supporting intervention, for example, providing them with coping strategies, would reduce the appearance of psychological disorders [51,52]. Any such intervention should consider age, since it is a negative element in acceptance, rumination, refocus on planning, positive reappraisal, and putting into perspective strategies. On the contrary, we cannot affirm the existence of gender differences, simply a higher tendential difference in women in positive refocusing. Although in this case, the small number of men in the sample, which however, is also a faithful reflection of the characteristics of the nursing profession, should be kept in mind.

The results further showed that there were different profiles depending on the characteristics of the COVID-19 context, perceived threat, and whether or not someone close was COVID-19 positive. Authors such as Araujo et al. [28] or Eslami et al. [29] have already shown that the types of coping strategies used in a given situation vary depending on individual traits and experiences as well as the characteristics of the situation, which is in consonance with our study. This study also showed how COVID-19 contexts, characterized by high perceived threat, are associated with the more frequent use of “negative” coping strategies, such as rumination, catastrophizing, or other-blame, and with more somatic symptoms, anxiety/insomnia, social dysfunction, and depression. This information could be oriented toward the prevention of occupational risks which in future similar scenarios would help nurses develop positive coping strategies preventing health problems [27,28,29] and thus improving their physical and mental well-being. It would be of interest for future studies to explore our hypotheses to see whether they are confirmed in other sociogeographic settings and in other health professionals such as doctors, physiotherapists, etc. Future studies could also analyze the characteristics of work done by nurses at this time. As the questionnaire evaluated individual strategies, specifically, nine cognitive strategies for coping with negative situations, no strategies were included that were related to the workplace or social context in which they work.

Finally, a series of limitations should be pointed out, including the fact that variables such as health condition before the pandemic were not taken into consideration. Since the effects on health symptoms are greater in individuals with previous pathologies, future studies should find out their possible influence. In addition, as the study was cross-sectional, we could not find out whether the threat levels and coping strategies are maintained in the long term, as long as the COVID-19 diagnoses are maintained.

## 5. Conclusions

The current healthcare crisis has demonstrated the importance of adaptation and coping strategies for the general population and healthcare professionals. Nurses have been especially exposed to the difficult circumstances surrounding the pandemic, and nothing ensures that these circumstances will not be repeated. Furthermore, stress and uncertainty are constants in the routine of nursing care, and therefore, identification of coping strategies that provide better results is a useful exercise for the profession. Given that self-blame, rumination, catastrophizing, and other-blame are not beneficial strategies, it would be of interest to design interventions that inhibit those strategies and promote those that are beneficial, such as acceptance, positive refocusing, planning, positive reappraisal, or putting in perspective.

## Figures and Tables

**Table 1:** Coping strategies by sex. Independent samples *t*-test.

CERQ	Male			Female			*t*	*p*	Mean Dif.	SE Dif.	95% CI Mean Dif.		Cohen’s *d*
	N	M	SD	N	M	SD					Lower	Upper	
SB	49	7.73	2.46	302	7.41	2.25	0.91	0.363	0.32	0.35	−0.37	1.01	0.14
AC	49	14.38	3.02	302	14.09	2.58	0.72	0.471	0.29	0.40	−0.50	1.09	0.11
RU	49	11.83	2.89	302	12.41	3.50	−1.09	0.275	−0.57	0.52	−1.61	0.46	−0.16
PF	49	12.46	3.50	302	13.49	3.33	−1.97	0.049	−1.02	0.51	−2.03	−0.00	−0.30
RP	49	14.57	2.83	302	14.75	2.88	−0.40	0.684	−0.18	0.44	−1.05	0.69	−0.06
PR	49	15.14	3.17	302	15.24	3.09	−0.21	0.831	−0.10	0.47	−1.04	0.83	−0.03
PP	49	13.93	3.49	302	14.51	2.79	−1.28	0.201	−0.57	0.44	−1.44	0.30	−0.19
CA	49	7.85	2.60	302	8.22	3.00	−0.81	0.418	−0.36	0.45	−1.26	0.52	−0.12
OB	49	9.83	4.08	302	9.68	4.35	0.23	0.816	0.15	0.66	−1.15	1.46	0.03

Note. CERQ_SB = Self-blame, CERQ_AC = Acceptance, CERQ_RU = Rumination, CERQ_PF = Positive refocusing, CERQ_RP = Refocus on planning, CERQ_PR = Positive reappraisal, CERQ_PP = Putting into perspective, CERQ_CA = Catastrophizing, CERQ_OB = Other-blame.

**Table 2:** Perceived threat by COVID-19 and coping strategies. Correlations and descriptive statistics.

CERQ Subscales		SB	AC	RU	PF	RP	PR	PP	CA	OB
Perceived Threat	Pearson’s r	0.190	0.087	0.338	−0.016	0.061	−0.138	−0.069	0.397	0.149
	*p*-value	<0.001	0.105	<0.001	0.760	0.256	0.010	0.200	<0.001	0.005
	Upper 95% CI	0.289	0.190	0.427	0.088	0.164	−0.034	0.036	0.481	0.250
	Lower 95% CI	0.087	−0.018	0.241	−0.121	−0.044	−0.239	−0.172	0.305	0.045
32.95 (6.27)	M (SD)	7.45 (2.28)	14.13 (2.65)	12.33 (3.42)	13.34 (3.37)	14.72 (2.87)	15.23 (3.10)	14.43 (2.89)	8.17 (2.94)	9.70 (4.31)

Note: SB = Self-blame, AC = Acceptance, RU = Rumination, PF = Positive refocusing, RP = Refocus on planning, PR = Positive reappraisal, PP = Putting into perspective, CA = Catastrophizing, OB = Other-blame.

**Table 3:** Coping and health strategies. Correlation matrix.

CERQ Subscales	GHQ-28	Somatic Symptoms	Anxiety/Insomnia	Social Dysfunction	Depression
Self-blame	Pearson’s r	0.183	0.226	0.084	0.207
	*p*-value	<0.001	<0.001	0.116	<0.001
	Upper 95% CI	0.282	0.323	0.187	0.305
	Lower 95% CI	0.080	0.124	−0.021	0.105
Acceptance	Pearson’s r	−0.065	0.034	−0.169	−0.078
	*p*-value	0.223	0.527	0.001	0.146
	Upper 95% CI	0.040	0.138	−0.065	0.027
	Lower 95% CI	−0.169	−0.071	−0.269	−0.181
Rumination	Pearson’s r	0.333	0.406	0.090	0.190
	*p*-value	<0.001	<0.001	0.093	<0.001
	Upper 95% CI	0.423	0.490	0.193	0.289
	Lower 95% CI	0.237	0.315	−0.015	0.087
Positive refocusing	Pearson’s r	−0.119	−0.133	−0.227	−0.300
	*p*-value	0.026	0.013	<0.001	<0.001
	Upper 95% CI	−0.015	−0.028	−0.125	−0.202
	Lower 95% CI	−0.221	−0.234	−0.324	−0.392
Refocus on planning	Pearson’s r	−0.019	0.010	−0.189	−0.166
	*p*-value	0.718	0.857	<0.001	0.002
	Upper 95% CI	0.085	0.114	−0.086	−0.062
	Lower 95% CI	−0.124	−0.095	−0.288	−0.266
Positive reappraisal	Pearson’s r	−0.191	−0.180	−0.319	−0.373
	*p*-value	<0.001	<0.001	<0.001	<0.001
	Upper 95% CI	−0.088	−0.077	−0.221	−0.280
	Lower 95% CI	−0.290	−0.279	−0.410	−0.460
Putting into perspective	Pearson’s r	−0.080	−0.132	−0.147	−0.218
	*p*-value	0.136	0.013	0.006	<0.001
	Upper 95% CI	0.025	−0.028	−0.043	−0.116
	Lower 95% CI	−0.183	−0.233	−0.248	−0.316
Catastrophizing	Pearson’s r	0.336	0.457	0.203	0.301
	*p*-value	<0.001	<0.001	<0.001	<0.001
	Upper 95% CI	0.426	0.536	0.301	0.393
	Lower 95% CI	0.240	0.370	0.100	0.202
Other-blame	Pearson’s r	0.171	0.245	0.098	0.179
	*p*-value	0.001	<0.001	0.065	<0.001
	Upper 95% CI	0.271	0.341	0.201	0.279
	Lower 95% CI	0.067	0.144	−0.006	0.076
	M (SD)	9.63 (4.82)	10.61 (5.00)	8.01 (3.20)	2.20 (3.42)

Note: CERQ = Cognitive Emotion Regulation Questionnaire, GHQ = General Health Questionnaire.

**Table 4:** Coping strategies and health by cluster. Descriptive statistics.

CERQ		C1	C2	C3	C4
Self-blame	M	7.27	7.81	7.14	7.60
	SD	2.49	2.14	2.15	2.44
Acceptance	M	14.14	14.26	14.15	13.80
	SD	2.64	2.39	2.78	2.99
Rumination	M	11.27	13.10	12.09	12.76
	SD	3.72	3.04	3.67	2.78
Positive refocusing	M	13.28	13.20	13.43	13.64
	SD	3.72	3.25	3.21	3.42
Refocus on planning	M	14.60	14.78	14.78	14.70
	SD	3.19	2.62	2.98	2.71
Positive reappraisal	M	15.79	14.84	15.38	14.94
	SD	2.74	3.13	3.25	3.17
Putting into perspective	M	15.07	14.32	14.23	14.06
	SD	3.04	2.74	2.70	3.31
Catastrophizing	M	6.99	9.09	7.32	9.72
	SD	2.76	2.86	2.39	3.15
Other-blame	M	8.74	10.06	9.06	11.76
	SD	4.18	4.31	3.86	4.69
**GHQ-28**		**C1**	**C2**	**C3**	**C4**
Somatic symptoms	M	7.58	11.95	7.78	11.38
	SD	4.32	4.42	3.85	5.33
Anxiety/insomnia	M	7.99	12.80	9.05	12.94
	SD	4.63	4.52	4.18	5.05
Social dysfunction	M	7.59	8.69	7.02	9.18
	SD	2.48	3.59	2.79	3.37
Depression	M	1.57	2.79	1.39	3.54
	SD	2.77	3.55	2.29	5.03

## Data Availability

The data that support the findings of this study are available from the corresponding author upon reasonable request.
